# Behavioural biomarkers of typical Rett syndrome: moving towards early identification

**DOI:** 10.1007/s10354-016-0498-2

**Published:** 2016-08-11

**Authors:** Christa Einspieler, Michael Freilinger, Peter B. Marschik

**Affiliations:** 1 Research Unit iDN, interdisciplinary Developmental Neuroscience, Institute of Physiology, Center for Physiological Medicine, Medical University of Graz, Harrachgasse 21/5, 8010 Graz, Austria; 2 Department of Paediatrics and Adolescent Medicine, Medical University of Vienna, Vienna, Austria; 3 Center for Neurodevelopmental Disorders, Department of Women’s and Children’s Health (KIND), Karolinska Institutet, Stockholm, Sweden

**Keywords:** Infant, Movement, Verbal behavior, Speech, Phonation, Säugling, Bewegung, Verbales Verhalten, Sprechen, Phonation

## Abstract

The dynamic course of Rett syndrome (RTT) is still said to begin with a period of apparently normal development although there is mounting evidence that individuals with RTT show behavioural peculiarities and abnormalities during their infancy. Their spontaneous general movements are abnormal from birth onwards. Normal cooing vocalisation and canonical babbling (if at all required) are interspersed with abnormalities such as proto-vowel and proto-consonant alternations produced on ingressive airstream, breathy voice characteristics, and pressed or high-pitched vocalisations. The gestural repertoire is limited. Certain developmental motor and speech-language milestones are not at all acquired or show a significant delay. Besides abnormal blinking, repetitive and/or long lasting tongue protrusion, and bizarre smiling, there are already the first body and/or hand stereotypies during the first year of life. We are currently on a promising way to define a specific set of behavioural biomarkers pinpointing RTT.

## Zusammenfassung

Die vorherrschende Lehrbuchmeinung über die Pathogenese des Rett-Syndroms (RTT) ist immer noch so, dass die frühe Entwicklung annähernd normal verläuft, obwohl Ergebnisse aus Elterninterviews, -fragebögen, aber auch aus Videoanalysen belegen, dass Patientinnen mit RTT bereits im Säuglingsalter auffällig sind. Ihre Spontanbewegungen („general movements“) sind nicht altersadäquat und qualitativ abnormal; ihre frühen Vokalisationen (z. B. das Gurren und das kanonische Lallen) sind zumindest intermittierend auffällig mit alternierenden Protovokalen und Protokonsonanten bei einwärts strömender Luft, hauchiger, gepresster oder hoher Stimme; das Repertoire an kommunikativen Gesten ist limitiert; bestimmte motorische, soziokommunikative und sprachliche Meilensteine werden nicht oder nur mit großer Verzögerung erworben. Neben einseitigem Blinzeln, wiederholter und lang anhaltender Zungenprotrusion sowie bizarrem Lächeln kommt es bereits im ersten Lebensjahr zum Auftreten von Stereotypien des Körpers oder der Hände. Mit diesen Befunden nähern wir uns derzeit der Definierung eines spezifischen Spektrums an frühen Auffälligkeiten, die typisch für das RTT sein könnten.

## Rett syndrome and the analysis of its early behaviour

Mutations in the X‑linked gene encoding Methyl-CpG-binding protein 2 (MeCP2) account for 95–97 % of cases of Rett syndrome (RTT, MIM312750), a genetic disorder affecting neurodevelopment, predominantly in females [1, 2]. Among the key features of typical RTT are (a) stereotyped hand movements coinciding with a regression in purposeful hand use, and (b) a regression in expressive language [2–4]. The dynamic course of RTT is said to involve a period of apparently normal early development followed by a profound neurological regression and subsequent stabilization or partial recovery [2–4]. This view, however, contrasts with mounting evidence of behavioural peculiarities and abnormalities observable already during the first months of life (e. g. [5, 8]).

RTT is usually diagnosed around 3 years of age [9], and being a rare disease, there are hardly any possibilities except retrospective data analyses to track down the pathways of various developmental domains before the assumed loss of previously acquired functions. Retrospective analyses comprise (a) parental interviews and questionnaires including parental diaries and medical histories; and (b) home video analyses. Both methodological approaches have strengths and weaknesses. Limitations of interviews/questionnaires are (i) a long time lag between the period of interest and the interview/questionnaire, (ii) a memory bias of parents with affected children and (iii) the lack of parental training in the observation of certain developmental features, especially when it comes to qualitative aspects of behaviours [10, 11]. The analysis of family videos (recorded at a time when the caregivers were not aware of their child’s disorder) is a more reliable option to focus on prediagnostic behaviour. It allows the detailed description of observable phenomena and behavioural peculiarities. But, one must never conclude that the absence of a certain behaviour is definite as home videos are not standardised, and parents tend to record situations which they would like to keep as pleasant memory. Nevertheless, some peculiar behaviours or even abnormalities—often unrecognised by parents—do not escape the eye of the camera [10, 12].

## The motor domain

### Spontaneous general movements

The assessment of the quality of early spontaneous movements, also known as the Prechtl assessment of general movements (GMs), has repeatedly proven to be a valuable tool in detecting early markers for neurodevelopmental disorders (e. g. [13–16]). GMs are generated by a neural network, the central pattern generators (CPGs), which are most likely located in the brainstem [14, 17]. GMs involve the entire body in a variable sequence of neck, arm, trunk and leg movements. Supraspinal projections activate, inhibit and most importantly, modulate the CPG activity, as does the sensory feedback [14, 17]. Reduced modulation of the CPG results in less variable (i. e. abnormal) movements and indicates neonatal or young infant’s compromise (e. g. [13, 14, 16]).

Already the very first reports on viewing some of the family videos (later used for a more detailed analysis [7, 18]) revealed the impression that infants later diagnosed with RTT moved in a jerky and uncoordinated way [19]. Detailed analysis including GM assessment [14] demonstrated that their GMs were clearly impaired (Table 1). Within the first 2 months of life, infants later diagnosed with RTT had already poor repertoire GMs [7, 18, 20, 21]; the sequence of their movement components was monotonous and the intensity, speed, and range of motion lacked the normal variability [13, 14].

**Table 1:** Prominent signs and peculiarities in infants later diagnosed with typical Rett syndrome

	Rate of occurrence	References
Poor repertoire general movements (birth–2 months after term)	9/11 (82 %)	[7, 18, 20, 21]
Absent or abnormal fidgety movements (3–5 months after term)	14/14 (100 %)	[7, 18, 20, 21]
Significant delayed in or no achievement of sitting alone, pulling to stand or walking alone	20–50 %	[20, 25, 27, 28]
Touching objects with extended fingers rather than manipulating them	14/24 (58 %)	[7, 20, 30]
Hand stereotypies	>50 %	[7, 19, 20, 29–32]
Asymmetric opening of the eye lid after a blink	10/18 (56 %)	[7]
Frozen, bizarre, inadequate smile	>35 %	[6, 7, 20, 26]
Repetitive or long-lasting tongue protrusion	13/21 (62 %)	[7, 20, 29]
Delay and/or abnormalities^a^ in cooing vocalisation and canonical babbling	>50 %	[8, 20, 33, 34, 37]
Limited use of gestures	13/16 (80 %)	[11, 20, 26, 38, 39]
Delay in speaking the first (proto-)word	>80 %	[8, 20, 34, 39]
Insensitivity to pain	Anecdotic	[20, 26, 27, 30]

^a^Proto-vowel or proto-consonant alternations produced on ingressive airstream, breathy voice characteristics and pressed or high-pitched vocalisations.

In typical development a new pattern of GMs, the so-called ‘fidgety movements’ emerge at the beginning of the 3^rd^ month [13, 14]. Fidgety movements are small movements of the neck, trunk and limbs in all directions and of variable acceleration; they last until the end of the 5^th^ month when intentional movements become predominant [14, 22]. If fidgety movements are present and normal in their quality, infants will very likely develop normally [13, 23]. None of the 16 infants with a later diagnosis of RTT (reported in the literature) ever showed normal fidgety movements; their fidgety movements were either absent or abnormal, i. e. jerky and too slow, or jerky and abrupt [7, 18, 21].

In addition to these early movement abnormalities, subtle disturbances of muscle tone [5–7, 20, 24–26] and tremulous movements [7] were already observable during the first months of life.

### Gross motor performance

In one of our first retrospective video analyses of infants later diagnosed with RTT we found that all 22 participants had the head centred in the midline by the 3^rd^ month. When they had reached the end of their 6^th^ month, almost all infants were able to roll over, and 3/22 infants were already able to sit without support [7]. Similar results were reported in the so far largest retrospective study based on parental interview. Nearly all of the 542 females with typical RTT were reported to have had acquired gross motor milestones such as rolling and sitting with support [27]. However, other more advanced gross motor behaviours such as sitting alone (80 %), crawling (69 %), pulling to stand (62 %), or walking alone (53 %) were less likely acquired [27], and if so, a significant developmental delay was reported [20, 25, 27, 28].

### Fine motor performance

Witt-Engerström [29] discussed RTT as affecting the voluntary arm and hand movements even before hand skills were lost. Although >73 % of infants and toddlers with a later diagnosis of RTT acquired—albeit most of them delayed—fine motor behaviours such as reaching, transferring an object, pincer grasping or finger feeding [27], video analyses demonstrated that 14/24 infants (58 %) hardly manipulated toys but just touched them with extended fingers [7, 20, 30].

### The first body and hand stereotypies

Repetitive limb and trunk movements, and small twitching movements of eyes and mouth were among the behavioural peculiarities described for the first year of life [19, 31]. In our own study we observed two 5‑month-old girls with stereotyped side-to-side body rocking while simultaneously shacking or nodding the head [7].

Meticulous video analyses revealed that the following hand stereotypies were clearly recognisable within the first months of life: repetitive opening and closing of the hand(s), excessive hand patting, twisting movements of the wrists and arms, uni- or bilateral repetitive pronation of the hand with simultaneous dorsiflexion of the wrist or repeated bringing of the palmar sides of both hands together, raising both hands and separating them [7, 19, 20, 29–32]. By contrast to later stages of RTT, hand stereotypies in the first year of life are still interspersed with a variety of normal and purposeful hand movements and postures [7, 20, 30].

## The speech-language and socio-communicative domain

Typically developing children begin to communicate through pre-linguistic vocalisations, eye gaze, responsive smiling and gestures to express their wants and needs before they produce their first (proto-)words. Audio-video analysis but also parental questionnaires revealed that infants later diagnosed with RTT had besides motor abnormalities also deviant behaviours in their developing speech-language and socio-communicative domain (Table 1).

### The eyes and the smile

In the so far largest sample analysed from home videos [7], almost all 1‑ to 6‑month-old infants later diagnosed with RTT were visually interested and demonstrated adequate visual pursuit. Apart from their early visual interest, a considerable number of infants (41 %) had strabismus, and more than half of the infants (56 %) had asymmetric opening of the eye lid after a blink (lasting even up to a few minutes) or asymmetric closing of the eye lid during a blink [7].

Although parental interviews revealed social smiling in almost all infants with a later diagnosis of RTT [27], several home video analyses displayed a frozen, bizarre or inadequate smile during the first months of life [6, 7, 20, 26]. Also, frequent and partly long-lasting tongue protrusions were observed from the first month onwards [7, 20, 29].

### Early pre-linguistic vocalisations

Although parental interviews revealed that the majority of infants with a later diagnosis of RTT had developed early speech-language milestones such as cooing (93 %) or babbling (95 %) [27], detailed analysis of family videos demonstrated that this was not the case for all individuals [8]. As mentioned above, both methodological approaches have their limitations: (a) parents are naïve observers in describing (pre-)linguistic phenomena, (b) they might not remember details of certain behaviours happening a few years earlier, and (c) the linguistic corpus is always defective as it never covers the whole set of vocalisations present. Hence, we need to be cautious in drawing conclusions *if* a certain developmental milestones was age-adequately acquired. On the other hand, audio-video analyses have the strength to focus on the complexity, composition and quality of early vocalisations (including low-level descriptors [33]). In this respect, it turned out that a considerable number of infants later diagnosed with typical RTT [8, 34] but also with the preserved speech variant [34–36] presented abnormal vocalisations on inspiratory airstream. Especially from 3 months onwards, normal cooing vocalisation was interspersed by proto-vowel or proto-consonant alternations produced on ingressive airstream, breathy voice characteristics, and pressed or high-pitched vocalisations [8, 33–37].

The majority of infants with available recordings on canonical babbling demonstrated again an interspersed pattern of typical and atypical babbling [8, 34]. These deviant characteristics in early vocalisations of infants later diagnosed with RTT could be accurately identified by 400 participants of a listening experiment. The rating of canonical babbling led to a more accurate differentiation between typically developing infants and infants later diagnosed with RTT as compared to cooing vocalisations [37].

### Gestures

First gestures such as demonstrating and/or passing an object, index finger pointing, and reaching towards the caregiver usually emerge around 10 to 12 months of age. Although the age of onset of the first gestures was adequate in infants with a later diagnosis of RTT, the gestural repertoire was limited [11, 20, 26, 38, 39]. Symbolic gestures such as nodding the head were observed in a very limited number of infants later diagnosed with RTT. It remained however open if this gesture conveyed a meaning or was rather a perseverative motor pattern [11, 39]. The gestural repertoire was limited in terms of number but also in its usage, i. e. the pragmatic functions mainly observed were attention to self, requesting an action or object, or imitation [11, 39].

### The first (proto-)word

According to audio-video analyses of family videos hardly any infant later diagnosed with typical RTT spoke proto-words (3/19; 16 %) [8, 20, 34, 39], and none of them produced word combinations during the second year of life [8].

## Other early signs

Apart from frequently reported early feeding difficulties [5, 6, 27, 31], insensitivity to pain attracted parents’ and the professional observer’s attention [20, 26, 27, 30, 35]. Infants with a later diagnosis of RTT hardly reacted when hurt.

## Conclusion

Individuals with RTT may achieve certain developmental milestones such as standing and walking alone, babbling, using gestures and/or speaking the first words. A series of studies have, however, shown that these milestones bear important information if we look beyond the yes/no (achieved/not achieved) dichotomy. Qualitative deviances, among them abnormal GMs and/or abnormalities in cooing and babbling account for CPG involvement in the brainstem [7, 14, 17, 40, 41]. Our understanding of the ways in which *MECP2* impacts early brain development is constantly evolving. Deficiencies in both organisation and refinement of early neural circuits, altered neurogenesis, and aberrant cell signalling might explain some of the early developmental abnormalities (for review see [42]).

Although Table 1 provides a list of early signs in infants later diagnosed with RTT, we know that certain deviations are shared by individuals with other neurodevelopmental disorders. Further (multicentre) studies are essential (a) to sort out the specific set of early behavioural biomarkers pinpointing RTT, and (b) to elucidate interconnectivity and (the lack of) modulation of CPG activities, which are crucial for the very early (dys)function of motor and vocalisation behaviours.
